# The Application of Transesophageal Echocardiography in Mitral Valve Repair With Tendon Reconstruction

**DOI:** 10.3389/fsurg.2020.599746

**Published:** 2020-12-16

**Authors:** Li Zhou, Ji-wei Gu, Yun Wang, Jing-jing Ye, Fang Wang, Ting-ting Wang, Bo Jiang, Li-sha Na

**Affiliations:** ^1^Department of Cardiac Functions Examination, General Hospital of Ningxia Medical University, Yinchuan, China; ^2^Department of Cardiovascular Surgery, General Hospital of Ningxia Medical University, Yinchuan, China

**Keywords:** transesophageal echocardiography, mitral valvuloplasty, tendon reconstruction, prediction, cardiovascular surgery

## Abstract

**Objective:** To investigate whether tendon reconstruction during mitral valve repair can be effectively guided by transesophageal echocardiography (TEE), using the mid-esophageal bi-commissure view, bicaval view and the aortic valve–mitral valve transition short-axis view.

**Methods:** A total of 40 patients that underwent mitral valve repair with artificial tendineae were recruited. Before the operation, conventional transthoracic echocardiography was used to determine whether mitral valve repair would be possible. Following intraoperative anesthesia, two-dimensional and three-dimensional TEE reconstructions were used to assess the state of the valve and tendon and to make a repair plan.

**Results:** TEE accurately diagnosed single functional tendon rupture and predicted single artificial tendon implantation in 88% of cases (23/26). TEE accurately diagnosed single functional tendon rupture and predicted the implantation of two artificial tendons in 100% of cases (4/4). TEE accurately diagnosed two or more functional tendon ruptures and predicted the implantation of two artificial tendons in 100% of cases (5/5). The length of the tendon cord predicted by TEE (2.45 ± 0.15 mm) was not significantly different (*P* > 0.05) from the length of the cord that was actually implanted (2.31 ± 0.11 mm). TEE also accurately predicted the size of the annuloplasty ring in 86% of cases (33/38), with differences of 2 mm or less compared to the size of the ring that was actually implanted.

**Conclusion:** Both the mid-esophageal bi-commissure view, bicaval view and the short-axis view of the aortic valve–mitral valve transition can reduce the difficulty of tendon reconstruction by helping to determine what length of tendon and what size of artificial annulus are required.

## Introduction

The severity of mitral valve regurgitation caused by mitral valve prolapse largely depends on the degree of change in the valve morphology, the number and location of the ruptured tendons, and the extent to which the annulus is enlarged (1). The success of mitral valve repair depends on the repair of the valve, the implantation of artificial tendon cords, and the choice of an appropriate forming ring. Both single and multiple artificial tendons (primarily constructed from Gore-Tex), can be implanted, and the implantation point is chosen according to the needs of the patient. The use of artificial tendons has greatly expanded the applications of valve repair and has become a standard method of repairing mitral valve prolapse (2). However, it can be challenging to determine what length of tendon is required, and a tendon that is too short or too long will seriously affect the shaping of the valve. This study explored whether artificial tendon length and annulus size could be determined with transesophageal echocardiography (TEE), using the mid-esophageal bi-commissure view, bicaval view and the short-axis view of the aortic valve–mitral valve transition.

## Materials and Methods

### Research Objects

The patient group comprised 40 patients that underwent mitral valve repair with tendon implantation between January 2016 and December 2019 at the Department of Cardiovascular Surgery of the General Hospital of Ningxia Medical University. The patient group included 13 women and 27 men, aged 22–61 years (mean: 51.3 ± 13.3 years). Transthoracic echocardiography and auxiliary examinations were used to diagnose all patients with pure mitral valve prolapse and moderate or severe regurgitation. The prolapse was diagnosed as one and/or two valvular part of or the whole mitral valve protruding to the left atrium during systole, reaching or 2 mm exceeding the level of annulus connecting line. Pure mitral valve prolapse was defined as that the lesion existed only in the mitral valve and no organic lesions of other valves were combined. Abnormal tendons were one of the primary causes of valve prolapse in the patient group. Patients were excluded from the study if they had rheumatic mitral valve disease or ischemic mitral valve regurgitation, or if they had already undergone mitral valve repair without artificial tendons, or patients with organic valvular diseases requiring surgical treatment. There were six cases of moderate mitral regurgitation (16%), 24 cases of moderate-to-severe regurgitation (63%), and eight cases of severe regurgitation (21%). In addition, 15 patients experienced complications from Medium and above tricuspid regurgitation, 10 patients experienced complications from atrial fibrillation, and two patients required left coronary artery bypass grafting to be performed at the same time.

### Instruments and Methods

#### Instruments

The following instruments were used: a Philips iE33 color Doppler ultrasound machine with an S5-1 transthoracic probe and an X7-2t three-dimensional (3D) TEE phased array probe (Philips Healthcare, Andover, MA, USA).

#### Image

##### Acquisition and Analysis

**Figure 1:**
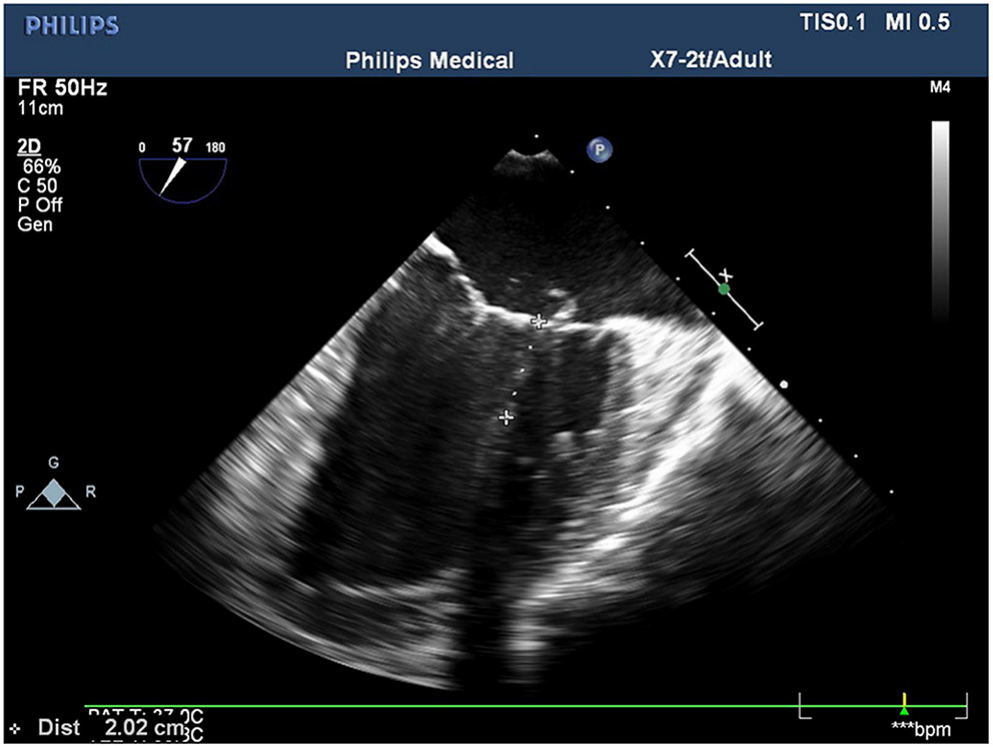
Length of the tendons measured at position P1 on the mid-esophageal bicaval view.

**Figure 2:**
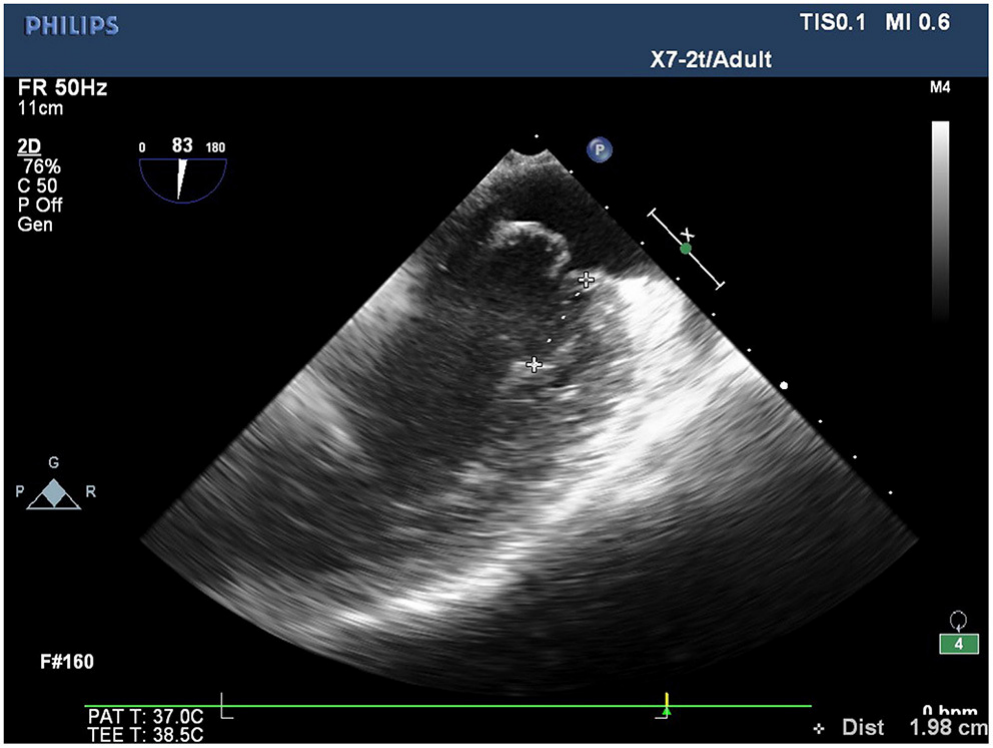
Length of the tendons measured at position A1 on the mid-esophageal bicaval view.

**Figure 3:**
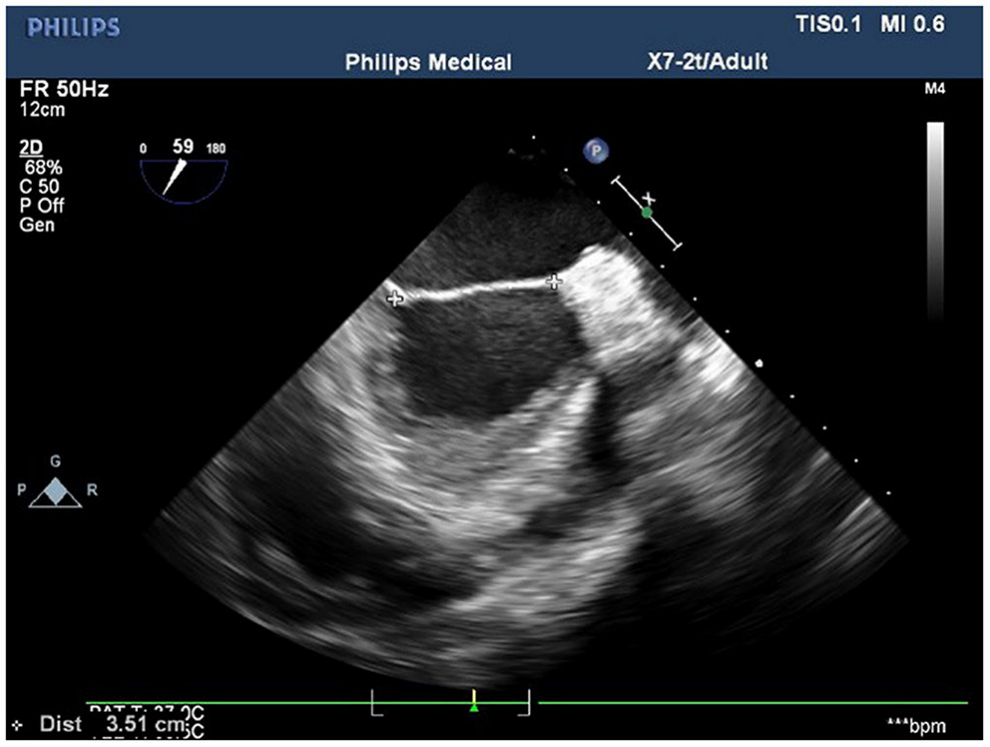
The measurement of the distance between the fiber triangles at 50° on the short-axis view of the aortic–mitral valve transition.

Conventional transthoracic echocardiography was used to determine each patient's heart size and cardiac function, evaluate the location and cause of mitral valve lesions, and establish whether shaping would be possible (Table 1). Following general anesthesia for angioplasty, 2D TEE was used to determine the valve's thickness, echo, and length and the subregion of the prolapsed leaflets, the subvalvular tendon, and the papillary muscles. The nature and extent of the valve and tendon lesions were determined using 3D TEE. 2D-TEE scan clearly showed P1-A2-P3 and bilateral junction on the mid-esophageal bi-commissure view at about 60–70°. This section could show the P1 chordae tendineae, and the mid-esophageal bi-commissure view at 80–100° can clearly show the A1-P3. The length of the valve adjacent to the ruptured chordae tendineae at position A1 or P1 to the top of the connected papillary muscle was measured at the end of diastole, that is, the predicted length of the implanted chordae tendineae (see Figures 1, 2). The aortic valve disappeared when the probe was pulled out about 50° on the short axis at the bottom of the heart, and the mitral valve was deflected to the side of the mitral valve so that the mitral valve loomed into a line, and its length was measured as the distance between the fibrous triangles on both sides (see Figure 3). After the valve was repaired with an edge-to-edge suture and a resection of the partial flail leaflet, a double-ended needle and spacer were used to fix the artificial tendon to the tip of the papillary muscles and the edge of the valve. A valve measuring device was used to measure what size of annulus was required and to further fine-tune the length of the tendon after an artificial annulus of the appropriate size was implanted. The anterior and posterior leaflets lay on the same mating plane and had the largest possible mating area. If the water injection test was successful, the surgeon who was blinded to the experiments then used a fine ruler to measure the actual length of the implanted artificial tendon. If multiple tendons were implanted or the loop method was used, the average tendon length was calculated using the actual length of each implanted tendon. Immediately after the cardiopulmonary bypass, TEE was used to determine whether the valve repair procedure had been successful. The following grading standards were used to measure post-operative valve regurgitation: level 0: a regurgitation area of 0–1 cm^2^; level 1: a reflux area of 1–3 cm^2^; level 2: a reflux area of >3 cm^2^; level 3: a reflux area of >3 cm^2^ accompanied by a reverse of blood flow in the left atrium; level 4: all characteristics of level 3 accompanied by systolic pulmonary venous blood flow. All patients that successfully underwent angioplasty had a post-operative reverse flow grade of 0–1, a height of mitral valve alignment >8 mm (3), no obvious valve stenosis, and no forward movement of the mitral valve anterior systole (i.e., systolic anterior motion). The same physician performed all ultrasound examinations and monitored the patients during echocardiography.

**Table 1:** General patient clinical data and routine ultrasound measurements.

**Item**	***N***
Male	27
Ages (years)	54.8 ± 9.8
BMI	23.9 ± 2.6
Smoking	10
COPD	0
Hypertension	13
Diabetes	2
Pulmonary hypertension	5
Post-operative blood transfusion	17
LVEDD (mm)	59.3 ± 6.9
Left ventricular end-systolic internal diameter	37.6 ± 4.9
Left atrium front and back path	48.8 ± 8.2
EF (%)	65.4 ± 5.1
Stroke volume (ml)	115.6 ± 31
CPB (min)	149.3 ± 49.5
ACC (min)	99.5 ± 38.5
Mitral annuloplasty ring (mm)	31.1 ± 2.3
Ventilator hours (h)	28.6 ± 73

### Statistical Methods

All data in this study were analyzed using the SPSS 17.0 statistical software (IBM Corp., Armonk, NY, USA). Measurement data were expressed as mean ± standard deviation (x ± s), and a paired *t*-test was used to compare the tendon cord length predicted by TEE with the actual length of the implanted tendons. Correlation coefficients and Bland-Altman analysis were used to evaluate the accuracy of assessment of the subregions of the mitral valve prolapse, chordal length and annulus diameter. *P* < 0.05 was considered statistically significant.

## Results

In 40 patients undergoing mitral valve repair with artificial tendon implantation, preoperative measurements were taken to determine the average left ventricular end-diastolic diameter (62.5 ± 6.9 mm) and the average left ventricular end-systolic diameter (46.1 ± 5.3 mm). The average ventricular ejection fraction was 62.2 ± 3.6%. In addition to artificial tendon implantation, five patients received end-to-end suture, 27 patients underwent resection of the partial flail leaflet, and six patients underwent other surgical procedures. The valves of 38 patients were successfully reshaped; these patients experienced no mitral regurgitation during the 6 months of follow-up period. In two patients, valve repair was not successful: two patients underwent mitral valve replacement after experiencing moderate regurgitation after valve repair.

The different diagnoses and measurements made by TEE were compared with those made during the operation.

### Assessment of the Subregions of the Mitral Valve Prolapse

For the 38 patients with mitral valve prolapse that were included in the study, the results of TEE identified 24 cases of posterior leaflet prolapse, nine cases of anterior leaf prolapse, and five cases of mixed anterior and posterior leaf prolapse. Intraoperative diagnosis demonstrated that the diagnostic accuracy of TEE was 99% (36/38). For cases of single subregional prolapse, TEE had a diagnostic accuracy of 99% (24/25): Whereas, 25 cases were diagnosed intraoperatively, TEE diagnosed 24 (17 in the posterior P2 area, 2 in the P3 area, 4 in the frontal A2 area, and 1 in the A3 area). For cases of prolapse in two or more subregions, TEE had a diagnostic accuracy of 88% (14/16): Whereas 16 cases were diagnosed intraoperatively, TEE diagnosed 14 (7 in the P2 and P3 areas, 5 in the A2 and P2 areas, 1 in the P3 and C2 areas, and 1 in the A3, C2, and P3 areas). The case that TEE identified as being in the P3 and C2 areas was intraoperatively diagnosed as being in the P3 area only, and the case that TEE identified as being in the A3, C2, and P3 areas was intraoperatively diagnosed as being in the A3 and C3 areas only. Overall correlation coefficient: 0.927; *P* = 0.000.

The tendon *pathological conditions by TEE identification, predicted number, and length of the tendons to be implanted compared with intraoperate*.

**Figure 4:**
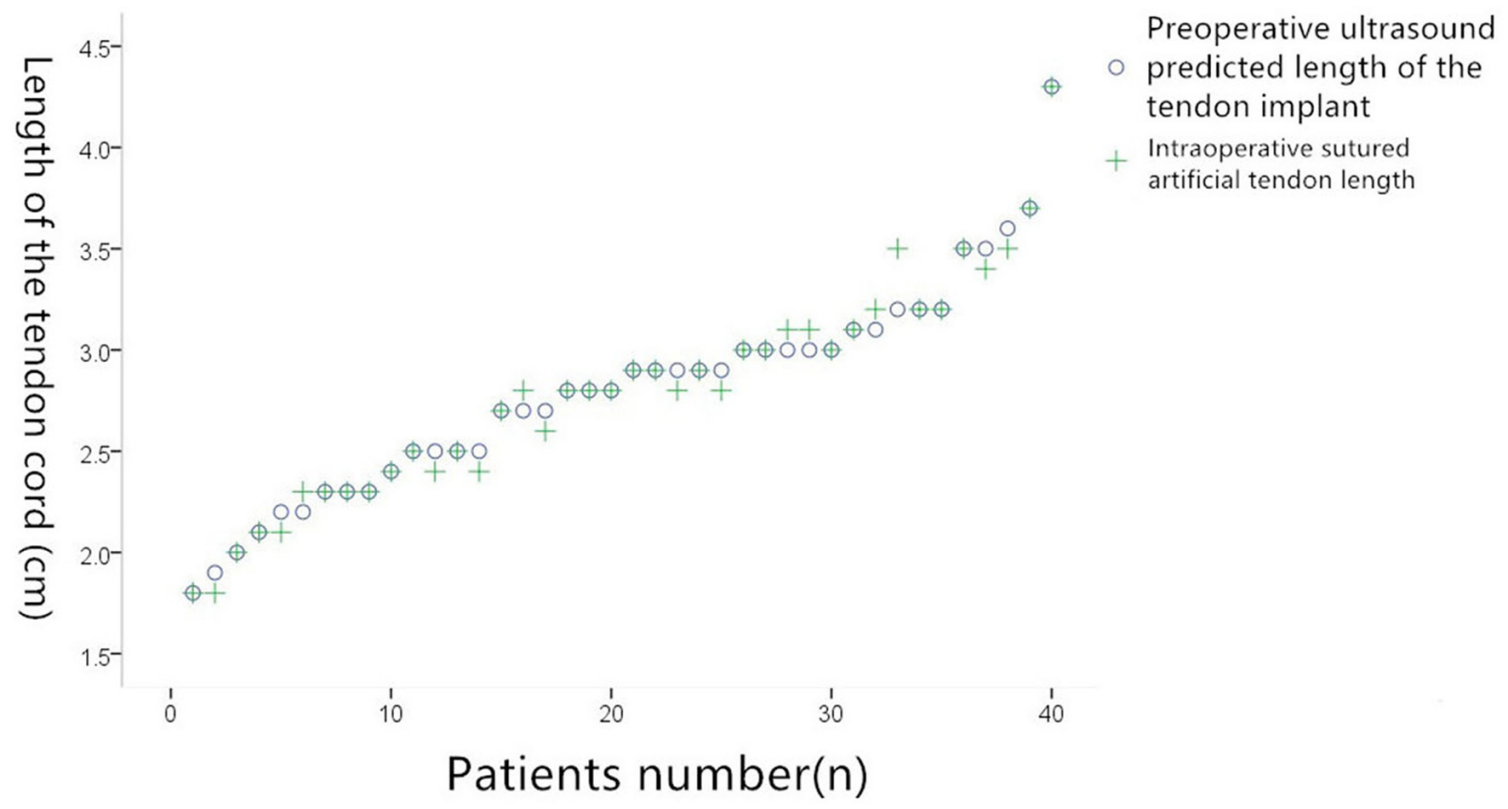
Comparison of preoperative ultrasound predicted suture tenosynovial length and actual suture tenosynovial length during surgery.

In the 38 patients in whom artificial tendon implantation was successful, TEE identified 28 cases of single functional tendon rupture, five of which were cases of root fracture and above and five of which were lengthy. By contrast, intraoperative investigation identified 26 cases of single tendon rupture, with 23 cases that required one tendon to be implanted and three cases that required two tendons to be implanted. TEE had an accuracy of 88% (23/26). TEE predicted that two tendons would need to be implanted in four cases; this prediction was confirmed for all four cases by intraoperative investigation, giving TEE an accuracy of 100% (4/4). TEE identified five cases of two or more functional tendon ruptures and predicted that two artificial tendons would need to be implanted in each case. Intraoperative investigation confirmed this (where the tendons were implanted using the loop technique in two cases), giving TEE an accuracy of 100% (5/5). Overall correlation coefficient: 0.922; *P* = 0.000. According to the results of the paired *t*-test, there was no significant difference (*P* > 0.05) in the average length of the tendons that TEE predicted would be required (2.45 ± 0.15 cm) and the average length of the tendons that were actually implanted (2.31 ± 0.11 cm) (Figure 4).

### Assessment of the Size of the Required Annulus

**Figure 5:**
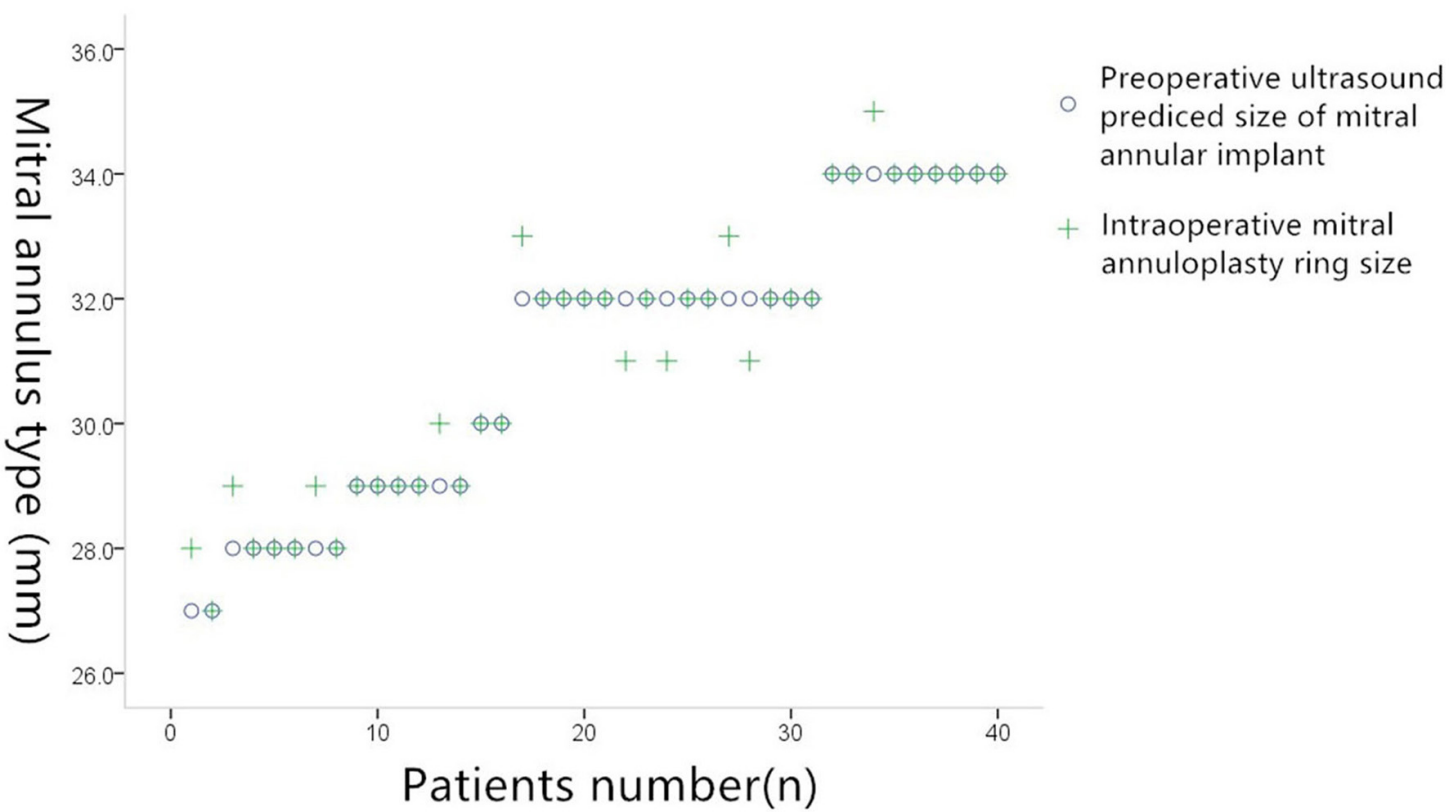
Comparison of preoperative ultrasound prediction of implanted mitral annuloplasty and actual intraoperative implantation of mitral annuloplasty.

The annulus size predicted by TEE was identical to the actual size in 33 out of 38 cases, giving TEE an accuracy of 86% (33/38). Overall correlation coefficient: 0.977; *P* = 0.000. In the five cases in which TEE predicted a different size, the difference between the predicted and actual sizes was ±2 mm. The exact differences in these five cases were as follows: In three cases, the TEE model returned 30 mm whereas the valve detector model returned 31 mm; in one case, the TEE model returned 29 mm whereas the valve detector model returned 27 mm; and in one case, the TEE model returned 32 mm whereas the valve detector model returned 31 mm (Figure 5).

## Discussion

It is difficult to control the length of artificial tendons during mitral valve repair (4), and it is often necessary to adjust the tendons' length repeatedly during the operation to test their shaping effect. Mitral valve repair is a challenging operation with high technical requirements and a long operation time, and even a small deviation from the required tendon length can cause the valve to fail. TEE has been widely recognized to have clinical applications for mitral valve repair. Both 2D and 3D TEE have their own advantages, and, when used together, they complement each other and provide an indispensable tool that can make the operation smoother. Whereas, 2D TEE can clearly display the thickness, length, and echo of the mitral valve and accurately judge the valve's texture and flexibility (5), 3D TEE provides a dynamic bird's eye view of the mitral valve, which is equivalent to the surgical field of view and allows the clinician to evaluate the valve lesions and formulate a surgical plan (6). Unlike 2D TEE, 3D TEE can handle the non-planar characteristics of the mitral valve annulus and can accurately diagnose cases of mitral valve prolapse that are not easily detected by 2D TEE, such as cases of prolapse at the leaflet junction. In addition, 3D TEE can display the size of the prolapse range, laying the foundation for mitral valve formation. By contrast, the imaging principle affects 2D TEE judgment of tendon lesions; this can lead to the misidentification of lengthened or ruptured tendons. Only operators with significant experience can accurately determine the location and number of tendon ruptures using 2D TEE (7). Finally, 3D TEE can measure tendon length with greater sensitivity than 2D TEE can: Although the number and location of tendon ruptures can be clearly displayed with 2D TEE, offline reconstruction of the mitral valve structure is still necessary to determine the length of the tendons. This is a complex and time-consuming process that is difficult to carry out during surgery, where tendon length is a valuable reference point for the surgeon. When performing mitral valve repair, surgeons often determine the required length of the artificial tendon on the basis of the A1 and P1 segments, which are the least likely to have lesions. If the lesion involves the A1 and P1 segments, however, the required tendon length is determined on the basis of the normal valve leaflet segment adjacent to the lesion (8). The 2D mid-esophagus series of slices can show all areas of the mitral valve leaflet (9): The P1, A2, and P3 segments and the junction between the two chambers can be seen at ~60–70°, and the A1 and P3 subregions are visible at ~80–100°. The two groups of papillary muscles and the tendons that are connected to them are also visible. Because the 2D ultrasound measurement method is simple and fast, it has the potential to aid diagnosis during an operation. We measured the length of the normal tendon at the A1 or P1 segment adjacent to the broken tendon, comparing it with the actual length of the tendon implanted during surgery, in order to explore whether this section can provide a reference value to determine the length of the tendon to be implanted. We found that the length of the tendon cord as measured by 2D TEE (2.85 ± 0.15 mm) was highly consistent with the length of the actual tendon cord implanted during surgery (2.91 ± 0.11 mm). This suggests that 2D TEE can be used effectively in mitral valve repair.

The mitral valve provides another important reference point and can reduce the difficulty and length of the operation. The mitral annulus is a saddle-shaped 3D structure that is divided into an anterior annulus and a posterior annulus. The two ends of the aortic curtain attach to both sides of the anterior lobe at the fiber triangles. Previous studies have found that the size of the forming ring predicted by TEE measurements of the distance between the fiber triangles is basically equivalent to the size measured during surgery (10). In this study, the size of the annulus was predicted by using TEE to measure the distance between the fiber triangles on the short-axis view of the aortic–mitral valve transition, imaged at ~50°. Compared with the size of the actual implanted annulus, TEE had an accuracy rate of around 95%, with differences in measurement of 2 mm or less. This finding is consistent with the literature (11). In summary, 2D and 3D TEE technologies can play an important role in mitral valve repair with artificial tendon implantation: They can be used to evaluate the overall and local lesions of the mitral valve, determine a surgical plan, and repair the valve morphology. The mid-esophageal bicaval view and the short-axis view of the aortic–mitral valve transition can be used to determine what length of tendon and what size of artificial annulus are required. This can make the tendon reconstruction operation less difficult.

## Limitations

The relevant tendon and other submitral valve structures are typically located in the far field of TEE imaging, and the high-frequency probe used in TEE has lower definition in the far field than in the near field. This limitation will be addressed by the development of new TEE technologies that can more accurately measure tendon length and that are better suited for clinical applications.

## Data Availability Statement

The original contributions presented in the study are included in the article/supplementary material, further inquiries can be directed to the corresponding author/s.

## Ethics Statement

The studies involving human participants were reviewed and approved by Ethics Committee General Hospital of Ningxia Medical University. The patients/participants provided their written informed consent to participate in this study. Written informed consent was obtained from the individual(s) for the publication of any potentially identifiable images or data included in this article.

## Author Contributions

LZ and L-sN conceived the idea and conceptualized the study. J-wG and YW collected the data. J-jY and FW analyzed the data. T-tW drafted the manuscript, then BJ reviewed the manuscript. All authors read and approved the final draft.

## Conflict of Interest

The authors declare that the research was conducted in the absence of any commercial or financial relationships that could be construed as a potential conflict of interest.

## References

[1] Suh YJ Lee S Chang BC Shim CY Hong GR Choi BW Utility of cardiac CT for preoperative evaluation of mitral regurgitation: morphological evaluation of mitral valve and prediction of valve replacement. Korean J Radiol. (2019) 20:352–63. 10.3348/kjr.2018.0350 30799566 PMC6389816

[2] Vahanian A Urena M Ince H Nickenig G. Mitral valve: repair/clips/cinching/chordae. EuroIntervention. (2017) 13:AA22–30. 10.4244/EIJ-D-17-00505 28942383

[3] Kagiyama N Shrestha S. Echocardiographic assessment of mitral regurgitation. J Med Ultrason (2001). (2020) 47:59–70. 10.1007/s10396-019-00971-1 31446501

[4] Matsui Y Kubota S Sugiki H Wakasa S Ooka T Tachibana T. Measured tube technique for ensuring the correct length of slippery artificial chordae in mitral valvuloplasty. Ann Thorac Surg. (2011) 92:1132–4. 10.1016/j.athoracsur.2011.03.111 21871323

[5] Miyatake K Okamoto M Kinoshita N Izumi S Owa M Takao S. Clinical applications of a new type of real-time two-dimensional Doppler flow imaging system. Am J Cardiol. (1984) 54:857–68. 10.1016/S0002-9149(84)80222-2 6486038

[6] Wei J Hsiung MC Tsai SK Ou C-H Chang C-Y Chang YC The routine use of live three-dimensional transesophageal echocardiography IM mitral valve surgery: clinical experience. Eur J Echocardiogr. (2010) 11:14–8. 10.1093/ejechocard/jep173 19933520

[7] Mori M Yoshimuta T Ohira M Yagi M Sakata K Konno T. Impact of real-time three-dimensional transesophageal echocardiography on procedural success for mitral valve repair. J Echocardiogr. (2015) 13:100–6. 10.1007/s12574-015-0255-3 26223699

[8] Huang HL Xie XJ Fei HW Xiao XJ Liu J Zhuang J. Real-time three-dimensional transesophageal echocardiography to predict artificial chordae length for mitral valve repair. J Cardiothorac Surg. (2013) 8:137. 10.1186/1749-8090-8-137 23721153 PMC3674909

[9] Wang XF Li ZA Cheng TO Deng YB Zheng LH Hu G. Clinical application of three-dimensional transesophageal echocardiography. Am Heart J. (1994) 128:380–8. 10.1016/0002-8703(94)90492-8 8037106

[10] Shanewise JS Cheung AT Aronson S Stewart WJ Weiss RL Mark JB. ASE/SCA guidelines for performing a comprehensive intraoperative multiplane transesophageal echocardiography examination: recommendations of the American Society of Echocardiography Council for Intraoperative Echocardiography and the Society of Cardiovascular Anesthesiologists Task Force for certification in perioperative transesophageal echocardiography. Anesth Analg. (1999) 89:870–84. 10.1097/00000539-199910000-00010 10512257

[11] Anwar AM Soliman OI ten Cate FJ Nemes A McGhie JS Krenning BJ. True mitral annulus diameter is underestimated by two-dimensional echocardiography as evidenced by real-time three-dimensional echocardiography and magnetic resonance imaging. Int J Cardiovasc Imaging. (2007) 23:541–7. 10.1007/s10554-006-9181-9 17164985

